# Case of Acute Insanity; with Remarks

**Published:** 1839-09-14

**Authors:** Joseph B. Cottman

**Affiliations:** Resident Physician at the Philadelphia Hospital, Blockley


*Case of Acute Insanity; with Remarks.* Reported by JOSEPH B. COTTMAN, M. D., Resident Physician at the Philadelphia Hospital, Blockley.

J—— B——, æt. 35, gambler, entered the lunatic department of the hospital, May 29th, 1839, in a very excited state; can get very little of his anterior history at present; afterwards learned that he was born on the Eastern Shore of Maryland; was there a sailor; owned a boat; came to Philadelphia some three or four years ago, and sold it; a part of the money he loaned; with the other he commenced gambling; a short time after, his wife died; since that time, has been living with an abandoned woman; has children both by his wife and this woman; a few weeks ago he lost a large sum of money; immediately commenced reading the Bible, both day and night, until Tuesday evening, May 29th, when he became very much excited and noisy, broke the windows of his house, and attempted to destroy all within his reach; was taken by an officer, and brought to the hospital.

*Present State.* Patient is a large, muscular man; dark hair and eyes; expression wild; face flushed; pupils dilated; skin warm and dry over body; skin of head and face very hot; throbbing of carotids; pulse very much excited; answers some questions rationally, but will give no account of himself; when asked if he was an intemperate man, became very much enraged, and attempted to strike me; have since learned that he was very intemperate; at one time he prays earnestly, at another curses bitterly, and uses the most obscene language; he was so unmanageable, that the keeper was directed to put a strait-jacket on him, and strap him to his bedstead, to prevent him from doing himself or others injury; this enraged him the more, and in the absence of the keeper he made a successful attempt to break loose; having obtained his liberty, he inflicted a severe blow on the head of an assistant with an iron lock; he was, however, again secured; his head was then shaved, and one hundred American leeches applied to his temples, and behind the ears; drew about twenty ounces of blood; he soon became quiet, and talked more rationally; earnestly entreated to be loosened; said he knew well, and was sorry for, what he had been doing, but he was *mad;* thought at the time all around him were attempting to kill him, more particularly myself; determined to "fight his way;" is now fully convinced that none meant to harm him; prays much, and calls upon his Maker to save him; says he has been a great sinner.

*Treatment.* Ice to head, and confinement to a dark room.

*May 30th, A. M.* Slept none, but remained quiet during the night; is still much excited; begs constantly to be sent home; says "he is himself again;" pupils much dilated; skin warm; pulse frequent, bounding; bowels constipated; have not been open since he came in.

*Treatment.* Half an ounce of the *oleum ricini;* continue cold applications to head.

*P. M.* In much the same state; has been very noisy during the day; sometimes hallooes, at others persists in the most obstinate silence. On entering his cell, found him in this state, lying on the floor; could not obtain an answer from him; countenance fixed; expression anxious; pupils very much dilated; bowels have been open very freely since morning.

*Treatment.* Apply the same number of leeches to temples and behind the ears; afterwards continue applications of ice.

*31st, A. M.* Slept five hours; very quiet during the night, until about three o'clock this morning, when he became very noisy, broke the straps which confined him, and attempted to break open his cell-door; at present is very obstinate, takes no notice of any thing, cannot elicit an answer to any question proposed to him; leeches drew about eighteen ounces of blood; became quiet immediately after the application of leeches, and fell asleep.

*Treatment.* Continue cold applications.

*June 1st, A. M.* Slept none, but was very quiet during the night; expression anxious; pupils moderately dilated; pulse feeble; skin cool; bowels open once in the last twenty-four hours; eats nothing.

Continue treatment, and commence with three grains of calomel night and morning.

*2d, A. M.* Slept none; no change for the better since yesterday morning; positively refuses to eat any thing; has to be forced to do so.

Treatment the same.

*P. M.* Has been quiet during the day, until this afternoon, when he became very noisy for a short time; is now very quiet, and talks more rationally on some subjects than he has since he came in; thinks, however, that he is to be murdered; has a strong disposition to destroy himself; tongue coated with a white fur; pulse weak and feeble; no desire for food; bowels open once in twenty-four hours.

Continue treatment.

3*d*, *A. M.* Slept none; expression anxious; pupils slightly dilated; tongue coated; skin natural temperature; pulse feeble.

Continue remedies.

4*th*, *A. M.* Slept five hours and a half; appearance somewhat improved; observes objects, answers questions, still manifests a suicidal disposition; pupils natural, tongue cleaner, pulse rather stronger.

Treatment the same.

5*th*, *A. M.* Slept none; remained quiet through the night; this morning is in much the same state as yesterday; talks very little.

Treatment as before.

6*th*, *A. M.* Slept one hour; a little noisy during the night; is quiet, however, to-day, and talks rationally on many subjects; desired to have his strait-jacket off, and put on his clothes; was permitted to do so, on condition that he would conduct himself properly; walked about with the assistance of two men; feels very weak; much altered in appearance; very pale.

*Treatment.* Continue cold applications and calomel.

7*th*, *A. M.* Very quiet during the night; slept four hours; feels better; eat more yesterday than he has at any time since his entrance; to-day his strait-jacket was again taken off; very composed and quiet; asked for a Bible; sat up and read it for three or four hours; mouth is not affected by the calomel; pulse rather stronger; skin natural temperature; expression calm.

Continue remedies.

8*th*, *A. M.* Slept three hours; quiet this morning; his strait-jacket was taken off, and he was dressed; walked about; asked for a Bible, permitted to have it; read much during the day; eats very little; thinks some one wishes to poison him.

Continue treatment.

9*th*, *A. M.* Went to bed without his strait-jacket, as he was so quiet during the day; became noisy in the night; prayed much, and frequently quoted parts of the Bible; slept none; this morning has a determined expression on his face; fixes his eyes on an object, and looks at it steadily for five or ten minutes; is evidently more excited for having read the Bible so much for the last two days; it was therefore forbidden; permitted to get up and walk about.

*P. M.* In much the same state as morning; prayed much during the day; expression anxious; pupils dilated; pulse stronger and fuller.

*Treatment.* Apply fifty leeches to temples and behind the ears; continue cold applications and calomel.

10*th*, *A. M.* Slept two hours; became quiet immediately after the application of leeches, and remained so during the night; this morning is somewhat improved; eats very little.

11*th*, *A. M.* Slept four hours; rest disturbed; wakeful; appeared to dream much; awoke often and cried aloud, then prayed; still refuses to eat any thing cooked in the house; says it has poison in it.

12*th*, *A. M.* Slept three hours; quiet during the remainder of the night; no change since yesterday morning.

Continue treatment.

13*th*, *A. M.* Rested better last night; slept five hours; sleep less disturbed; this morning is more rational; expression better, and general appearance improved; has regained his strength somewhat; permitted to walk about by means of assistance.

*Treatment.* Discontinue cold applications; continue calomel.

14*th*, *A. M.* Slept five hours; refuses to eat; his mistress, who was now permitted to visit him, brought him food, which he eat; takes very little notice of her; talks much about his children; mouth is not yet affected by the calomel.

15*th*, *A. M.* Rested well; appetite improved; strength returning; is able to walk with very little assistance.

Continue calomel.

20*th*, *A. M.* Has been gradually improving since last note; rested well every night; slept from five to six hours; very quiet both night and day; walks about during the day without any assistance; eats better; general appearance very much improved; converses rationally; mouth is not yet affected by the calomel; since the 15th, has had a shower-bath occasionally at night.

Discontinue all treatment.

29*th*, *A. M.* Since last note, has been convalescing fast; appetite and strength has returned; sleeps well; colour has returned to his face, and his appearance is now natural; converses rationally on all subjects; for the last nine days has received no treatment; allowed a generous diet, and free exercise in the open air; at the earnest solicitations of his friends, he was discharged rather sooner than he otherwise would have been, though he was considered perfectly cured. I have since heard that he has shown no symptoms of insanity since he left the house.

*Remarks.* The immediate or remote cause of insanity is often very obscure; a variety of causes, both moral and physical, may have combined to aggravate this case. The excitement and depression of the gaming table, the precarious mode of life, and free indulgence in intoxicating liquors and sensual pleasures, had their effect; the immediate cause, however, of his attack, appears to have been the loss which he sustained. It is singular what different effects the same cause produces on different individuals. Some it would have incensed and enraged; others it would have driven to despair and suicide; but he bore it calmly, and commenced reading the Bible,—the only consolation. It brought, however, any thing else but consolation to his troubled mind, for at the expiration of two weeks he began to show symptoms of insanity, amounting, in a short time, to raving mania; it assumed very much the character of religious insanity, and one would have pronounced it such without knowing any thing of his anterior history.

On his entrance into the hospital, the plan of treatment adopted appeared to be clearly indicated, viz: to remove or lessen the diseased condition of the brain, on which we had reason to believe the insanity depended as its immediate cause; this was done by the local abstraction of blood, not carried, it is true, to the same extent as practised by many, yet sufficiently so to calm the patient. "The late Dr. Rush was of opinion that the evacuation of blood ought to be carried to a greater extent in madness than in any other acute disease whatever. From a patient sixty-eight years of age, he caused two hundred ounces of blood to be drawn in less than two months. Another patient of Dr. Rush's lost four hundred and seventy ounces, by forty-seven bleedings, in the course of seven months." Pinel and Esquirol, on the contrary, maintained that blood-letting was rarely indicated, and, when practised, tended rather to retard than hasten recovery. Pinel was persuaded that bleeding gave to the disease a tendency to degenerate into dementia. "M. Esquirol coincides with Pinel in the opinion that the diseased state on which mental derangement depends, is sometimes changed for the worse by bleeding. He says that he has seen madness increased after an abundant flow of the catamenia, and likewise after one, two, or three blood-lettings. In such cases, melancholy dejection has passed into furious madness." Much might be said on both sides of the question, and cases brought forward to verify it; but the judicious observations of M. Foville place the subject in the true point of view. He says:—"Without ever having pushed the employment of this remedy so far as Rush and Joseph Franck, I confess that it appears to me to be one of those, in the efficacy of which the greatest reliance may be placed." In addition to the bleeding, other antiphlogistic means were made use of, viz., the application of cold to the head, and the exhibition of small doses of calomel night and morning. The local blood-letting was, however, the most important therapeutic agent employed, for it produced an immediate amelioration of all his symptoms, whenever resorted to; about fifty ounces of blood were taken from this man during the whole course of treatment. After having reduced the severity of the attack by these means, Dr. Gerhard thought it advisable to commence with small doses of calomel night and morning; and although it produced none of its peculiar effect after having taken it for twenty days, yet I cannot but believe that it had an alterative influence, as well as its action in regulating the bowels, and aided materially in the cure.

Much obscurity still remains with regard to the treatment of insanity: although the efforts of Rush, Esquirol, Pinel, Pritchard, and many others, have given much valuable information on the subject, still much remains to be learned. The ill success which often attends the treatment of insanity, depends very much upon the want of appropriate institutions where this unfortunate class of individuals may be properly classified, and subjected to proper moral and physical treatment, combined with the medical. In an institution like this, comparatively little can be done in these respects; the place will not admit of it; the grounds around it are too limited; there is nothing to amuse, and very little to employ those who are disposed to work; yet as many, if not more cases of *recent insanity*, are cured here, as in any other similar institution. Efforts are now being made to establish a state institution for the insane, on a very liberal scale, and then we may have reason to hope that the *poor insane* of this state will be better provided for.

